# New onset diabetes in adulthood is associated with a substantial risk for mortality at all ages: a population based historical cohort study with a decade-long follow-up

**DOI:** 10.1186/s12933-017-0583-x

**Published:** 2017-08-15

**Authors:** Inbar Zucker, Tamy Shohat, Rachel Dankner, Gabriel Chodick

**Affiliations:** 1 Israel Center for Disease Control (ICDC) Ministry of Health, Tel Hashomer, Israel; 2 School of Public Health, Sackler Faculty of Medicine, Tel Aviv University, Tel-Aviv, Israel; 3 Unit for Cardiovascular Epidemiology, The Gertner Institute for Epidemiology and Health Policy Research, Tel Hashomer, Israel; 4 Maccabi Healthcare Services, Tel-Aviv, Israel

**Keywords:** Diabetes, All-cause mortality, Population based study

## Abstract

**Background:**

Diabetes has been reported to be associated with an increased relative risk for mortality, with estimates ranging from 1.1 to 2.1. Findings are inconsistent regarding modification of the risk by gender and by age. The aim of this study was to estimate the mortality risk associated with new-onset diabetes in adulthood, by age group and gender.

**Methods:**

From the database of a large health care provider, we identified 31,987 individuals diagnosed with diabetes during 2003–2005; and 162,656 individuals without diabetes, group-matched by age. We used Cox regression to calculate hazard ratios (HRs) and 95% confidence intervals (95% CIs) for overall mortality adjusted for age, gender, socioeconomic (SE) level, obesity, smoking and comorbidities at baseline.

**Results:**

During a median follow-up of 9.5 years, 4464 (14%) of persons with diabetes and 13,327 (8.2%) of those without died. Among persons with incident diabetes, the proportion of men, smokers, obese and patients of low SE level was higher, as was the prevalence of cardiovascular disease and renal impairment at baseline. Incident diabetes was associated with an adjusted HR for mortality of 1.38 (95% CI 1.32–1.43). Mortality HR for DM was comparable with hypertension (1.42; 1.37–1.46), smoking (1.65; 1.58–1.71) and atherosclerosis (1.40; 1.35–1.46). Diabetes associated mortality HR was somewhat higher among women 1.78 (95% CI 1.58–2.08) as compared with men 1.51 (95% CI 1.41–1.62).

**Conclusions:**

Incident diabetes in adults is associated with a substantial risk for mortality, especially in younger adults. Further efforts should be allocated to diabetes primary prevention.

**Electronic supplementary material:**

The online version of this article (doi:10.1186/s12933-017-0583-x) contains supplementary material, which is available to authorized users.

## Introduction

Diabetes is a chronic disease that can impair the normal function of many body systems. It affects almost 10% of the adult population in Israel and its prevalence is on the rise [1]. Numerous studies conducted in different countries have reported an association of diabetes with an increased risk for overall mortality, ranging from 1.1 to 2.07 [2–9]. Some studies found similar relative risk for men and women [2, 4, 6], while others reported a higher relative risk among women [3, 10]. The association between diabetes and mortality risk, according to age, also differed among studies. In most studies the highest relative risk was observed in the youngest age groups [4, 5], but in a study from Canada and the UK, the highest relative risk for mortality was observed in the 45–64 year age group [6]. Previous work conducted in Israel [4], with an average follow-up of 4.6 years, included both prevalent and incident diabetes cases, and did not address individual characteristics (except age and gender) that could confound the association between diabetes and mortality. In the decade since that work was published, major improvements have emerged in the field of diabetes care: new drugs were added to the therapeutic arsenal [11] and national quality control programs were developed [12]. The present study extends prior work by using a historical cohort design that enables comparing persons with and without diabetes, while controlling for differences in individual characteristics. The objectives of this study were: to provide an updated estimate of the risk for mortality associated with new-onset diabetes; to assess whether age and gender modify this risk and to assess the proportion of the risk that can be attributed to baseline co-morbidities.

## Methods

### Data sources

The study was carried out at Maccabi Healthcare Services (MHS) in Israel, which is the nation’s second-largest health maintenance organization, with coverage of 25% of the population. Since 1997, information on all members’ interactions (i.e., visits to outpatient clinics, hospitalizations, laboratory tests and dispensed medications) are downloaded daily to a central computerized database. Using this database, MHS developed and validated disease registries for major chronic diseases including diabetes, hypertension and heart disease. These registries served as the basis for the data extracted for this study [13]. The study was approved by the institutional review board of MHS.

### Study population

The MHS diabetes registry has been described in detail elsewhere [13]. Briefly, the registry was constructed in 1999 by an automated search in the MHS computerized databases, using the disease criteria suggested by the American Diabetes Association (ADA), including fasting blood glucose of ≥126 mg/ml, or a casual plasma glucose concentration of ≥200 mg/dl [14]. In accordance with the ADA guidelines [15] an HbA1c result of ≥6.5% in conjunction with a physician diagnosis of diabetes was later added to the registry inclusion criteria. The registry is continuously validated by computerized feedbacks from practitioners and was found to have 99.9% specificity [16]. As the registry does not include information about the type of diabetes we restricted the study population to age 35 years and older. The study cohort included all patients who entered the MHS diabetes registry at the age of 35 years or older during 2000–2005. The entry date to the registry served as the index date. The source population for establishing the comparison group comprised the diabetes-free members of MHS who were aged 35 years or older in 2003 and who were not included in the diabetes registry before the end of 2005. The index date of the diabetes-free group was set to January 1, 2003. Individuals who were diabetes-free were frequency-matched to cases by age (±1 year) in a ratio of 5:1. Active membership in MHS for at least 2 years prior to the index date was served as a study inclusion criterion; this was to enable an equal period for baseline data collection. Individuals who were diagnosed with cancer before the index date (n = 8560) or who were on dialysis treatment (n = 208) were excluded from the analysis.

### Data collection

Information of death is provided to MHS by the Israeli National Insurance Institute on a monthly basis. Follow up continued until death, leaving MHS or January 1, 2013, whichever came first.

#### Co-variates

Demographic variables included age at index date, gender and socioeconomic (SE) level, as assessed by the locality of residence. Based on the population census of 2008, the 210 localities in Israel were ranked on a 10-level ordinal scale according to the SE index of its population, as measured by 16 components including education, employment status and car ownership. For the current analysis, the localities were categorized into 3 groups: low (levels 1–4), medium (levels 5–7), and high (levels 8–10). Data on smoking status at baseline were retrieved from the electronic medical file. Smoking status was categorized as ‘current smoker’ and ‘not known to be a current smoker’, since the distinction between past-smoker and non-smoker was not accurate. For about 30% of the cohort smoking status was not recorded. We used two approaches to complete the missing data: (1) multiple imputations (MI), (2) expanding the time-window for smoking documentation to ±4 years from baseline. Individuals with any recording of active smoking within that period were classified as current smokers. These two approaches yielded similar results, therefore only the analysis using MI is presented. Data regarding baseline hypertension, cardiovascular disease (including ischemic heart disease, stroke and peripheral artery disease) and renal impairment were extracted from MHS medical registries. Since body weight was not routinely documented in the electronic medical record before 2005, weight data at baseline was available to only 10% of the cohort. Based on the relative stability of weight at adulthood [17], we performed a sensitivity analysis that included body weight documented within 5 years from baseline (available for 63% of the cohort). BMI was calculated based on weight and height, and obesity level was defined as: under/normal weight (BMI 14–24.9 kg/m^2^), overweight (25 ≤ BMI < 29.9 kg/m^2^) (mildly obese (30 ≤ BMI < 35 kg/m^2^) and severely obese (BMI ≥ 35 kg/m^2^).

### Statistical analysis

The χ^2^ tests for categorical variables (e.g. smoking, gender and hypertension) and the T test for continuous variables (e.g. age) were performed to determine differences in baseline characteristics between the groups. Cox proportional hazard regression models were used to estimate the risk for mortality associated with diabetes, by means of hazard ratios and their 95% confidence intervals. Model 1 was adjusted to age and gender. Model 2 included, in addition, the baseline co-morbidities: smoking status and SE level.

To examine whether age and gender modify the association between diabetes and mortality, the final model included the interactions terms of diabetes with age and gender.

Diabetes is often diagnosed indirectly throughout the work up of other, possibly fatal, disease. In such cases, diabetes is not what led to the death; rather, the fatal illness is what either caused the diabetes or led to its diagnosis. To minimize the possibility of this reverse causation bias, a sensitivity analysis was performed, in which the first year of follow-up was excluded.

All analyses were performed using IBM-SPSS software (IBM Corp. Released 2013. IBM SPSS Statistics for Windows, Version 22.0. Armonk, NY: IBM Corp).

## Results

The study comprised 31,987 incident diabetes patients and 162,656 individuals without diabetes. In the diabetes compared to the non-diabetes group, the proportion of men, smokers, obese individuals and persons of low SE level was higher, as was the prevalence of cardiovascular disease and renal impairment at baseline (Table 1).

**Table 1:** Baseline characteristics of the study groups

	With diabetes N = 31,987 n (%)	Without diabetes N = 162,656 n (%)	p*
Age at baseline (years) mean ± SD	58.1 ± 11.9	57.2 ± 11.6	<0.001
Females	14,258 (44.6)	86,961 (53.5)	<0.001
Comorbidity at baseline			
Hypertension	14,274 (44.6)	38,933 (23.9)	<0.001
Chronic renal failure	1984 (6.2)	5.843 (3.6)	<0.001
Atherosclerosis^a^	4666 (14.6)	11,795 (7.3)	<0.001
Heart failure	798 (2.5)	1421 (0.9)	<0.001
Arrhythmia	1405 (4.4)	4495 (2.8)	<0.001
Socio-economic level			<0.001
Low	4763 (15.3)	20,503 (12.9)	<0.001
Medium	18,343 (59.1)	90,761 (57.0)	<0.001
High	7944 (25.6)	47,940 (30.1)	<0.001
Current smoking	5599 (17.5)	25,967 (16.0)	<0.001
Under/normal weight (BMI 14–24.9 kg/m^2^)	3554 (12.4)	39,628 (30.5)	<0.001
Over weight (BMI 25–29.9 kg/m^2^)	10,533 (36.6)	53,931 (41.5)	<0.001
Mild obesity (BMI 30–34.9 kg/m^2^)	8950 (31.1)	26,336 (20.3)	<0.001
Severe obesity (BMI ≥ 35 kg/m^2^)	5706 (19.9%)	9973 (7.7%)	<0.001

* T test or Chi square test as appropriate

^a^ Including history of ischemic heart disease, stroke and peripheral artery disease

The median follow-up period was 9.5 (IQR 7.9–11.1) years, for the diabetes group, with a total of 294,204 person-years, and 10 (IQR 10–10) years, for the non-diabetes group, with a total of 1,522,185 person-years.

**Fig. 1:**
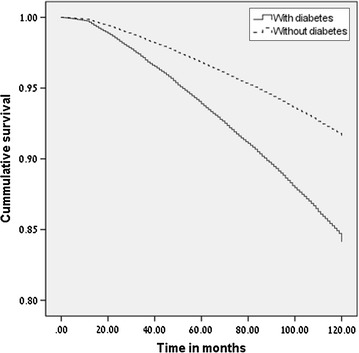
Survival plot according to diabetes status

During the follow-up period, 4464 (14%) of those with diabetes and 13,327 (8.2%) of those without diabetes died; mortality rates were 1.52 (95% CI 1.47–1.56) and 0.87 (95% CI 0.86–0.89) per 100 person-years respectively (Fig. 1).

Incident diabetes was associated with a 56% increased risk for mortality adjusted for age and gender (HR 1.56 95% CI 1.51–1.62). Age, male gender, smoking, low SE level and baseline comorbidity were all associated with an increased risk for mortality (Additional file 1: Table S1). In the fully adjusted model, the increased risk for mortality attributed to diabetes was reduced to 1.38 (95% CI 1.32–1.43) (Table 2). The sensitivity analysis of excluding death cases in the first year of follow up, yielded similar results (Table 2).

**Table 2:** Hazard ratio for mortality in individuals with new onset diabetes

	With diabetes deaths/total	Without diabetes deaths/total	Model 1 HR (95% CI)	Model 2 HR (95% CI)	Model 3
All	4464/31,987	13,327/162,656	1.56 (1.51–1.62)	1.38 (1.32–1.43)	1.61 (1.56–1.66)
Minimum 1-year follow-up	3772/31,132	12,212/160,040	1.46 (1.40–1.51)	1.28 (1.23–1.33)	1.58 (1.49–1.68)

Multivariate Cox regression analysis of people with new onset diabetes compared to people without diabetes. Model 1 adjusted for age and gender; Model 2 adjusted also for current smoking, SE level and baseline comorbidities; Model 3 included interaction term for diabetes with age (interaction term 0.98 for 1 year increase) and interaction term for diabetes with gender (1.12 for women)

**Fig. 2:**
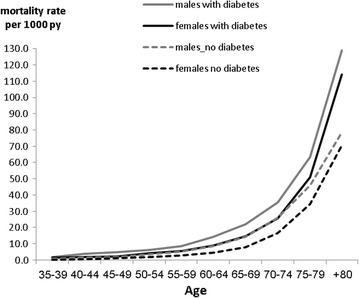
Mortality rate by diabetes status, gender and age

Age-specific mortality rates were higher among diabetic persons compared with non-diabetic persons in both genders and in all age groups. The mortality rates were higher in men than women in people with and without diabetes; (Fig. 2), but the diabetes associated HR for mortality was somewhat higher among women, (HR 1.78 95% CI 1.58–2.08) compared to men (HR 1.51 95% CI 1.41–1.62) (p for the interaction of diabetes and gender ≤ 0.001). In both genders, the diabetes associated HR for all-cause mortality decreased with older age (Table 3). The interaction of age with diabetes was statistically significant (p < 0.001).

**Table 3:** Hazard ratios for all-cause mortality of diabetics compared to non-diabetics by age and gender

	Men		Women	
	HR^a^	95% CI	HR^a^	95% CI
35–44 Diabetes n = 4071 No diabetes n = 23,477	1.87	1.38–2.54	2.69	1.34–5.38
45–54 Diabetes n = 9429 No diabetes n = 49,423	1.64	1.42–1.89	1.67	1.23–2.26
55–64 Diabetes n = 9020 No diabetes n = 44,381	1.47	1.33–1.63	1.55	1.28–1.88
65–74 Diabetes n = 6086 No diabetes n = 30,915	1.32	1.23–1.43	1.50	1.33–1.70
≥75 Diabetes n = 3381 No diabetes n = 14,460	1.14	1.07–1.21	1.24	1.14–1.34
All	1.51	1.41–1.62	1.78	1.58–2.08

^a^ HR adjusted to age, gender, current smoking, SE level and baseline comorbidities, and including interaction with age

## Discussion

In this historical cohort study we found that adults (aged 35 years and above) with newly diagnosed diabetes have an overall 38% increased risk for all-cause mortality compared to individuals without diabetes. This is higher than the 15% (95% CI 9–22%) increased risk for mortality reported by a Scottish population based study of individuals aged 35 and older with new onset diabetes, after adjusting for deprivation status and previous cardiovascular disease [18]. The population of that study was older than in our study (45% and 35% respectively were older than 65 years) which may explain the lower diabetes-associated mortality risk observed. Studies from the UK, Canada, and the US [3, 8, 19, 20] found that individuals with prevalent diabetes had an almost twofold increased risk for mortality compared to individuals without diabetes. Other studies conducted in Israel, Sweden and the Netherlands found lower relative risks, of <1.5 [2, 4, 9]. Comparing between studies is difficult due to differences in populations, in methodologies (the use of Cox regression vs. standardized mortality rates) and in the co-variates included in the multivariate analysis. In addition, due to the finding that age and gender modify the risk, overall estimates of the effect of diabetes on mortality are specific to study populations, since they are influenced by variations in population composition. The increased risk for mortality associated with diabetes was only partially explained by baseline risk factors (in the multivariate model the risk was attenuated by 18% from 1.56 to 1.38). Moreover, due to the insidious course of diabetes, its damage to the micro and macro vasculature often starts years before diabetes is diagnosed [21]. It is possible then, that some of the baseline comorbidities that we included in the multivariable model (e.g. ischemic heart disease and renal failure) were, in effect, caused by the diabetes, so they mediated the mortality risk and did not confound it. Therefore, the actual diabetes-related mortality risk may be somewhere between 1.38 and 1.56. Our finding of a higher diabetes associated risk for mortality among women as compared to men especially in the age group of 35–44 years is congruent with other reports [3, 4, 22–24]. Diabetes is a known risk factor for cardiovascular events [25] and was hypothesized to attenuate the female natural protection against cardiovascular complications [26, 27]. Similar to previous studies [3, 4, 22–24, 28] we report that the relative risk for mortality associated with diabetes decreased with age. This may be due to higher background mortality in older ages, or due to a survivor bias, i.e. a less severe course of diabetes that develops in those who survive to older age. Yet, even in those patients who were 75 years and older the risk remained pronounced, similar to a recent work from Taiwan [29]. Moreover, in the elderly, the lower relative risk translates to an addition of a larger number of deaths associated with diabetes, due to the high background mortality of this age group.

We found that the increased risk for mortality was already evident shortly following the diagnosis of diabetes as can be seen by the early separation of the survival curves (Fig. 1). Similarly, in the Cardiovascular Health Study, the diagnosis of diabetes among individuals aged 65 years and older was associated with a HR of 2.3 for mortality within the first 2 years after the diagnosis [10]. An immediate risk for mortality was also found among Danish males [30]. Taken together, this suggests that the mortality risk associated with the hyperglycemic state is not mediated solely through atherosclerotic pathways. The mortality associated with diabetes may be influenced by risk factors that are closely linked with hyperglycemia. One such possibility is insulin resistance, which was previously reported to be associated with cardiovascular events [31]. The finding that diabetes is associated with an increased risk for mortality at any age should be considered when prescribing medications for primary prevention of cardiovascular events, that have been reported to increase the risk for diabetes [32, 33]. This highlights the need to tailor treatments according to patient characteristics. The strengths of this study include the population-based design together with the usage of a large and valid electronic database with few dropouts. This minimizes the potential for selection and information biases. Another advantage is the comparison to individuals free of diabetes that belong to the same population as those with diabetes. Yet; our study has a few limitations. The data did not include information regarding the type of the diabetes; though by limiting the study population to patients diagnosed with diabetes at age 35 years and older the proportion with type 1 diabetes was presumably small [34]. The non-diabetes group may have included individuals with undiagnosed diabetes, as the disease can be asymptomatic for years. In a different project we found that 60% of MHS members at the age 35 years and older had their glucose tested at least once a year (data not published), so the prevalence of undiagnosed diabetes is expected to be low in this population. Similarly, it is possible that some individuals in the control group developed diabetes later on, causing some misclassification of the exposure, but the expected effect of this (if any) is to cause underestimation of the true risk. Another limitation was the fact that we did not have baseline BMI data and any data regarding important risk factors for mortality including physical activity, sedentary lifestyle and nutritional habits, which may differ between individuals with and without diabetes. In addition, we lacked data about the baseline level of important risk factors for mortality in diabetic patients including Hba1c, cholesterol and triglycerides [35, 36], and about the level of glucose or blood pressure control. In conclusion, our finding of 38% increased diabetes associated risk for mortality shows that despite the major advances in diabetes care and the introduction of new treatments, onset of diabetes still carries a significant increased risk for mortality. Furthermore, the increased risk was observed at all ages even among the elderly aged 75 years and above. These findings highlight the need to allocate resources to diabetes primary prevention and improved care in all ages.

## Additional file

**Additional file 1: Table S1.** Hazard ratios of multivariate Cox regression model.

## Abbreviations

HR: hazard ratio
CI: confidence interval
MHS: Maccabi Healthcare Services
ADA: American Diabetes Association
SE: socioeconomic
MI: multiple imputations
