# Supplementary material for: New onset diabetes in adulthood is associated with a substantial risk for mortality at all ages: a population based historical cohort study with a decade-long follow-up
Source: Cardiovasc Diabetol. 2017 Aug 15;16:105. doi: 10.1186/s12933-017-0583-x (PMC5558697; doi:10.1186/s12933-017-0583-x)
Supplement: Supplementary file 1 — Additional file 1: Table S1. Hazard ratios of multivariate Cox regression model. [file 12933_2017_583_MOESM1_ESM.docx]

**Additional file Table 1.** Hazard ratios of multivariate Cox regression model

|  | HR | 95% CI | P |
| --- | --- | --- | --- |
| Diabetes | 1.61 | 1.56-1.66 | <0.000 |
| Baseline age (one year increase) | 1.12 | 1.11-1.12 | <0.000 |
| Gender (female vs male) | 0.59 | 0.57-0.62 | <0.000 |
| Hypertension | 1.40 | 1.39-1.45 | <0.000 |
| Chronic renal failure | 1.27 | 1.21-1.33 | <0.000 |
| Atherosclerosis* | 1.40 | 1.34-1.45 | <0.000 |
| Heart failure | 2.22 | 2.08-2.37 | <0.000 |
| Arrhythmia | 1.39 | 1.32-1.46 | <0.000 |
| Current smoking | 1.65 | 1.58-1.71 | <0.000 |
| SE level (medium vs high) | 1.14 | 1.08-1.20 | <0.000 |
| SE level (low vs high) | 1.11 | 1.07-1.15 | <0.000 |
| Age*Diabetes interaction | 0.99 | 0.98-0.99 | <0.000 |
| Gender*Diabetes interaction | 1.12 | 1.02-1.20 | 0.002 |

*Including history of ischemic heart disease, stroke and peripheral artery disease.
